# Phosphate-solubilizing bacteria: a review of diversity, mechanisms, and applications in sustainable agriculture

**DOI:** 10.3389/fmicb.2026.1778470

**Published:** 2026-03-24

**Authors:** Xiaofei Sun, Xiaohang Tian, Mengxuan Jia, Xiaojuan Hu, Chenglin Zhang, Lina Zhao

**Affiliations:** College of Food and Bioengineering, Henan University of Science and Technology, Luoyang, China

**Keywords:** phosphate-solubilizing bacteria, diversity, acidolysis, enzymatic hydrolysis, plant-microbe interactions, PSB inoculant

## Abstract

Phosphorus (P) is an essential nutrient for plant growth but the global supply is limited, and over-reliance on chemical phosphate fertilizers can lead to soil pollution and ecological imbalance. Phosphate-solubilizing bacteria (PSB) can convert insoluble P in the soil into plant-available forms and enhance the P utilization efficiency of crops. The application of PSB can also improve the soil ecological environment and contribute to the sustainable development of agriculture. This review systematically summarizes the diversity and distribution of PSB. It comprehensively investigates the mechanisms through which PSB enhance soil P utilization efficiency, focusing on the following aspects: the acid-mediated solubilization of inorganic P, the enzymatic hydrolysis of organic P, the interactions between PSB and plant roots, and the interactions between PSB and rhizosphere microorganisms. Furthermore, recent advances in the development and application of PSB-based biofertilizers are also reviewed. Potential future research directions and the anticipated challenges within this field are also discussed, to develop innovative strategies for alleviating P deficiency in agricultural soils.

## 1 Introduction

Phosphorus (P) is a crucial nutrient for plant growth and serves as a limiting factor for crop yield (Bilal et al., 2021; Dokwal et al., 2021). It reported that phosphorus directly involved in nucleic acids synthesis, cell division and the growth of new tissues, and it also plays a crucial role in various processes such as plant photosynthesis, carbohydrate metabolism, energy production, redox homeostasis and signal transduction (Chouyia et al., 2022; Elhaissoufi et al., 2022; Lambers, 2022). However, only a small fraction (1.00–2.50%) of the total P in agricultural soils is in a form that is available for plant uptake (mainly HPO_4_^2–^ and H_2_PO_4_^–^) (Pan and Cai, 2023; Santoro et al., 2023). The slow diffusion of P in soil, coupled with its strong adsorption to mineral particles (such as Al, Fe and Ca), makes it difficult for plants to access sufficient P (Bilal et al., 2021; Richardson and Simpson, 2011). This leads to low crop yields under field conditions, severely limiting plant growth and agricultural development (Suleimanova Aliya et al., 2015). Given the agricultural demand for P, chemical phosphate fertilizers have been widely produced and applied globally to increase crop yields on the available arable land and feed the growing population (Akanmu et al., 2023; Wendimu et al., 2023). However, excessive use of phosphate fertilizers not only depletes the phosphorus retention capacity of the soil and increases the risk of phosphorus loss from the soil, but also affects soil fertility in the long-term use. Moreover, it may introduce toxic substances and contaminate the agricultural ecosystem (Li Z. et al., 2023; Wang et al., 2024). Therefore, it is imperative to identify environmentally compatible and economically viable alternatives to address P scarcity in agricultural soils (Pan and Cai, 2023).

According to a definition proposed by Malusá et al. (2012), PSB formulation is a biofertilizer containing one or more beneficial PSB strains and prepared using easy-to-use and economical carrier materials. Microbial inoculants have proven to be a cost-effective alternative to expensive and potentially environmentally harmful chemical applications, offering greater agronomic benefits than conventional fertilizers (Bi et al., 2019). The global biogeochemical cycles are largely driven by microorganisms, which play a key role in the conversion of organic matter into inorganic substances and make significant contributions to the turnover of soil materials (Sokol et al., 2022). Some of these microbes have been referred to as phosphate-solubilizing bacteria (PSB), with this group containing a more diverse set of metabolic capabilities to enhance the bioavailability of various insoluble P forms in soil (Mosimann et al., 2017). PSB play a critical role in the soil P cycle by converting insoluble P in the soil into plant-available forms, significantly improving crop yield and soil health (Pan and Cai, 2023). The discovery of PSB has provided an innovative solution to the global shortage of soil phosphorus resources. Its potential as a biofertilizer has received widespread attention. The biofertilizer made from PSB cannot only significantly improve the utilization efficiency of inorganic and organic fertilizers, but also effectively reduce the use of chemical fertilizers, thereby minimizing the negative impact of agricultural production on the environment (Wendimu et al., 2023). By optimizing soil nutrient utilization and improving crop nutrition, PSB offer a green solution for promoting sustainable agricultural development.

Currently, significant progress has been made both domestically and internationally in the research and application of PSB and their inoculants. For instance, bioformulated PSB strains (*Pantoea* sp. and *Pseudomona*s sp.) proved a great potential to enhance *Pisum sativum* growth (Anwar et al., 2019). Inoculation of rice with a combination of *Acinetobacter* sp. MR5 and *Pseudomonas* sp. MR7 increase the yield of grain and straw by 4–7% (Rasul et al., 2024). Tang et al. (2025) found that inoculation with PSB strain *P. asiatica* JP233 enhance the solubility of phosphorus in the soil and promoted the growth of tomatoes. However, challenges remain, including the need to increase PSB diversity, expand PSB strain resources, determine PSB characteristics, enhance inoculant stability, broaden the scope of applications, and evaluate long-term environmental impacts. Therefore, a deeper understanding of the mechanisms by which PSB promotes crop P uptake and the practical challenges of applying PSB-based biofertilizers is essential for their precise and effective utilization. This review provides a comprehensive overview of the diversity and distribution patterns of PSB. It elaborates on the biological mechanisms through which PSB enhance phosphorus acquisition in crops, including organic acid secretion for inorganic phosphorus solubilization, enzymatic mineralization of organic phosphorus, and synergistic interactions with plant root systems. The work further summarizes current progress in biofertilizer development using PSB and evaluates their potential for addressing phosphorus deficiency in agricultural soils. Additionally, the article discusses prospective research directions and identifies implementation challenges for deploying PSB-based solutions in sustainable agricultural practices.

## 2 Diversity and distribution of PSB

PSB are key functional microorganisms in soil ecosystems, and their diversity and distribution are influenced by variations in soil types and environmental conditions (Table 1). PSB are widely distributed across several bacterial phyla, including Proteobacteria, Firmicutes, and Actinobacteria, with several genera such as *Pseudomonas, Bacillus*, and *Rhizobium* demonstrating notable phosphate-solubilizing capabilities (Cumpa-Velásquez et al., 2021; Li H. P. et al., 2023). At the genus level, *Pseudomonas* spp. exhibit notable diversity, with strains such as *Pseudomonas fluorescens*, *Pseudomonas putida*, and *Pseudomonas taiwanensis* identified in Tabarak Forest in Tunisia (Amri et al., 2023). Similarly, species of the genus *Bacillus* demonstrate exceptional phosphate-solubilizing abilities, such as *Bacillus valismortis*, which was discovered in phosphate mines in Morocco (Ait-Ouakrim et al., 2023). Among the known PSB, *Pseudomonas*, *Bacillus*, and *Rhizobium* spp. are frequently regarded as the most potent phosphate solubilizers (Alori et al., 2017).

**TABLE 1:** Classification and distribution of phosphate-solubilizing bacteria.

Phylum	Genus	Species	Source	References
Proteobacteria	*Pseudomonas*	*Pseudomonas moraviensis, Pseudomonas fluorescens, Pseudomonas putida, Pseudomonas taiwanensis, Pseudomonas* sp. (L1, L3,L5,P1, P2, P3 and P4)*, Pseudomonas aeruginosa, Pseudomonas qingdaonensis, Pseudomonas azotoformans, Pseudomonas eucalypticola, Pseudomonas simiae, Pseudomonas guariconensis*	Sandy fluvo-aquic soil forest soil and agricultural soil rhizospheric regions of Shisham forests root system of tomato plants. Common rice fields rhizosphere of four legumes growing in acidic soils	(Amri et al., 2023; Bakki et al., 2024; Joshi et al., 2023; Kuang et al., 2024; Ríos-Ruiz et al., 2024; Samal and Sukla, 2024; Wang et al., 2022)
	*Pantoea*	*Pantoea agglomerans, Pantoea* sp. (L8)*, Pantoea brenneri, Pantoea agglomerans*	Forest soil and agricultural soil rhizospheric regions of Shisham forests soil samples of the Republic of Tatarstan roots and rhizosphere soil of two willow species	(Amri et al., 2023; Joshi et al., 2023; Koczorski et al., 2023; Suleimanova et al., 2023)
	*Stenotrophomonas*	*Stenotrophomonas maltophilia*	Forest soil and agricultural soil	(Amri et al., 2023)
	*Klebsiella*	*Klebsiella* sp. (L4 and T2), *Klebsiella pneumonia, Klebsiella quasipneumoniae, Klebsiella quasipneumoniae* subsp. *Similipneumoniae*	Rhizospheric regions of Shisham forests root samples of black pepper The kutch desert, Gujarat, India. common rice fields	(da Silva et al., 2024; Joshi et al., 2023; Kuang et al., 2024; Samal and Sukla, 2024; Yadav et al., 2023)
	*Rhizobium, Serratia*	*Rhizobium* spp.*, Serratia* spp.	Roots and rhizosphere soil of two willow species	(Koczorski et al., 2023)
	*Enterobacter*	*Enterobacter* sp.*, Enterobacter cloacae, Enterobacter bugandensis, Enterobacter asburiae, Enterobacter roggenkampii, Enterobacter* sp. (SS186)*, Enterobacter cloacae, Enterobacter bugandensis*	Root samples of black pepper rhizosphere of four legumes growing in acidic soils root samples of black pepper rhizosphere soil of *S. fruticosa* in two different saline environments: hypersaline plains and a deactivated salt mine The Kutch Desert	(da Silva et al., 2024; Ríos-Ruiz et al., 2024; Teles et al., 2023; Yadav et al., 2023)
	*Kosakonia*	*Kosakonia sacchari, Kosakonia pseudosacchari*	Root samples of black pepper, Baião city, state of Pará, Brazil	(da Silva et al., 2024)
	*Kushneria*	*Kushneria* sp. (SS102)	Rhizosphere of *S. fruticosa* in two different saline environments: hypersaline plains and a deactivated salt mine	(Teles et al., 2023)
	*Burkholderia*	*Burkholderia vietnamiensis, Burkholderia species strain* EIKU9	Common rice fields rhizospheric soil samples	(Kuang et al., 2024; Samal and Sukla, 2024)
	*Paraburkholderia, Acidovorax, Herbaspirillum, Novosphingobium, Pelomonas, Ralstonia, Roseateles, Segnochrobactrum*	*Paraburkholderia kururiensis* subsp. *Kururiensis, Acidovorax cattleyae, Herbaspirillum rubrisubalbicans, Novosphingobium humi, Pelomonas puraquae, Ralstonia mannitolilytica, Ralstonia* sp., *Roseateles depolymerans, Segnochrobactrum spirostomi*	Common rice fields in Luojiagang, Changde, China.	(Kuang et al., 2024)
	*Citrobacter, Escherichia, Raoultella, Leclercia, Salmonella, Acinetobacter, Ewingella*	–	Rhizospheric soil samples, in sodic-alkaline lowlands of the flooding pampa region	(Dip et al., 2024)
	*Nguyenibacter*	*Nguyenibacter* sp. L1	Rhizosphere soil surrounding wild Lespedeza bicolor plants, located in an acidic soil environment	(Li X. L. et al., 2023)
	*Cedecea, Delftia, Ensifer, Phyllobacterium, Sinorhizobium*	–	Rhizosphere soil of 15 years old poplar trees from a road verge	(Qingwei et al., 2023)
	*Nguyenibacter*	*Nguyenibacter* sp. L1	Acidic soil, Yingtan Red Soil Ecological Experimental Station, China	(Li X. L. et al., 2023)
Firmicutes	*Bacillus*	*Bacillus safensis, Bacillus paramycoides, Bacillus valismortis, Bacillus* spp*., Bacillus mycoides, Bacillus cereus, Bacillus paramycoides, Bacillus atrophaeus* (HL6)*, Bacillus subtilis* subsp. *Stercoris* (HG12)*, Bacillus* sp. (SS89)*, Bacillus cereus, Bacillus valismortis, Bacillus siamensis*	Sandy fluvo-aquic soil root system of tomato plants phosphate sludge roots and rhizosphere soil of two willow species root samples of black pepper rhizosphere samples rhizosphere soil of *S. fruticosa* in two different saline environments: hypersaline plains and a deactivated salt mine common rice fields phosphate mine rhizospheric soil samples	(Ait-Ouakrim et al., 2023; Bakki et al., 2024; da Silva et al., 2024; Koczorski et al., 2023; Kuang et al., 2024; Ma et al., 2024; Teles et al., 2023; Wang et al., 2022; Zapata et al., 2024)
	*Falsibacillus*	*Falsibacillus pallidus*	Sandy fluvo-aquic soil	(Wang et al., 2022)
	*Staphylococcus*	*Staphylococcus* sp. (L2 and T6)*, Staphylococcus* sp.	Rhizospheric regions of shisham forests rhizosphere of four legumes growing in acidic soils	(Joshi et al., 2023; Ríos-Ruiz et al., 2024)
	*Exiguobacterium*	*Exiguobacterium sibiricum* K1	Compost samples	(Kumari et al., 2023; Kumari et al., 2024)
	*Paenibacillus*	*Paenibacillus* spp., *Paenibacillus peoriae* (HG24)	Roots and rhizosphere soil of two willow species rhizosphere samples	(Koczorski et al., 2023; Ma et al., 2024)
	*Oceanobacillus*	*Oceanobacillus* sp. (SS94)	Rhizosphere soil of *S. fruticosa* in two different saline environments: hypersaline plains and a deactivated salt mine	(Teles et al., 2023)
	*Limosilactobacillus*	*Limosilactobacillus* sp. LF-17	Traditional dough fermentation starter	(Li et al., 2024)
	*Priestia*	*Priestia filamentosa* (HL3)*, Priestia aryabhattai*	Rhizosphere samples agricultural soil, domesticated under Cd stress conditions	(Ma et al., 2024; Pang et al., 2024)
Actinobacteria	*Brevibacterium*	*Brevibacterium frigoritolerans*	Phosphate mine	(Ait-Ouakrim et al., 2023)
	*Microbacterium*	–	Red soil, experimental forest farm	(Pang et al., 2024)
	*Kitasatospora, Micrococcus, Streptomyces*	*Kitasatospora* sp. (T1)*, Micrococcus* sp. (T4).*, Streptomyces* sp. (L6, L7,T3 and T5)	Rhizospheric regions of Shisham forests	(Joshi et al., 2023)
	*Brevibacterium*	*Brevibacterium frigoritolerans*	Phosphate sludge	(Ait-Ouakrim et al., 2023)
	*Streptomyces*	*Streptomyces venezuelae*		
		*Streptomyces* spp.	Roots and rhizosphere soil of two willow species	(Koczorski et al., 2023)
	*Paenarthrobacter*	*Paenarthrobacter nitroguajacolicus*	Rhizospheric soil	(Salimi et al., 2023)
	*Actinotalea*	*Actinotalea fermentans*	Common rice fields	(Kuang et al., 2024)
	*Arthrobacter*	–	Rhizospheric soil samples, in sodic-alkaline lowlands of the flooding pampa region	(Dip et al., 2024)
	*Cellulosimicrobium*	–	Rhizosphere soil of 15 years old poplar trees from a road verge	(Qingwei et al., 2023)
	*Micrococcus*	*Micrococcus indicus* EU-BRP-6	Rhizospheric soil of wheat, barley, and millets, the Himalayan mountains on the green slopes of the Shivaliks, Himachal Pradesh	(Kaur et al., 2024)

PSB are widely distributed in agricultural fields, forests, salinized soil (Li et al., 2025) and other various extreme environments (such as heavy metal-polluted soils and arid ecosystems) (Wang et al., 2025). PSB exhibit remarkable functional diversity and ecological adaptability across different soil types. For example, in a rice paddy field in Changde, China, *Paraburkholderia kururiensis* subsp. *Kururiensis* were found to exhibit strong phosphate-solubilizing activity, as strain showed transparent halos with a diameter greater exceeding 5 mm on the insoluble phosphate solid medium after cultivation, and indicating that the rice rhizosphere soil is a suitable habitat for efficient PSB strains (Kuang et al., 2024). Similarly, *Serratia* spp. and *Streptomyces* spp. are prevalent in the rhizospheres soil of willows in Sweden and Germany. These strains not only improve soil quality through phosphate solubilization mechanisms but also enhance plant nutrient acquisition via symbiotic interactions (Koczorski et al., 2023). PSB in extreme environments exhibit unique survival strategies. For example, in acidic soils, PSB display strong environmental adaptability. Fifty-five PSB strains were isolated from the rhizosphere soil of *Swida wilsoniana*, and soil physicochemical analyses confirmed that all samples were acidic (Gao et al., 2024). A novel aluminum phosphate-solubilizing bacterium, *Nguyenibacter* sp. L1, was also isolated from the rhizosphere soil of a native healthy *Lespedeza* plant in an acidic region (Li X. L. et al., 2023). Additionally, PSB have demonstrated resilience in high-salinity environments and can enhance the solubilization of insoluble phosphates by acidifying their surroundings. For example, *Arthrobacter* sp. isolated from saline lowlands in flood-prone areas of the Pampas grasslands in South America, exhibit significant salt tolerance and phosphate-solubilizing capabilities (Dip et al., 2024). PSB communities in different environments reflect the complex interactions between microorganisms and their environments. Future research should focus on the synergistic interactions within microbial communities in different soil types to fully determine their ecological functions.

PSB enhance soluble phosphorus availability in the environment through various pathways, including organic acid-mediated dissolution of inorganic phosphates and enzymatic mineralization of organic phosphates. The functional diversity of these bacteria is intrinsically linked to their genetic determinants: for instance, inorganic phosphate dissolution is principally governed by the *gcd* and *pqq* gene clusters through their mediation of organic acid biosynthesis, while organic phosphate mineralization is coordinately regulated by the *phoA* gene and *phoC* gene (Dai et al., 2020). Notably, the *gcd* genes are conventionally employed as functional markers for assessing phosphate-solubilizing capabilities in microbial systems. In a study of *Pseudomonas frederiksbergensis* JW-SD2, researchers observed that the presence of soluble phosphate didn’t induce significant upregulation of the *gcd* gene in this phosphate-solubilizing strain. The strain’s phosphorus-mobilizing capability varied depending on its production of specific organic acids and metabolic regulatory mechanisms. These findings suggest that *gcd* gene presence alone cannot fully explain phenotypic divergence in phosphate solubilization, as additional regulatory networks (e.g., phosphate starvation response pathways) and environmental adaptation mechanisms likely mediate this process through synergistic interactions (Hone et al., 2026). Neal et al. (2018) reported that genera possessing *phoC* gene, including *Caulobacter*, *Stenotrophomonas*, *Methylobacterium, Sphingomonas*, *Xanthomonas*, *and Pseudomonas*, prevalent in permanent grasslands and arable soils, exhibit acid phosphatase secretion capabilities facilitating organic phosphorus mineralization.

Current studies on PSB diversity have predominantly relied on conventional isolation methods (e.g., halo formation assays), which are limited to culturable microbial fractions and introduce inherent selection bias, while functional assessments via in vitro approaches (e.g., calcium phosphate solubilization assay) frequently overlook critical discrepancies between laboratory conditions and natural soil ecosystems. Subsequent research should integrate advanced methodologies to rigorously validate microbial functionality and assess potential ecological risks associated with strain applications.

## 3 Biological mechanisms by which PSB enhance phosphorus uptake by crops

**FIGURE 1:**
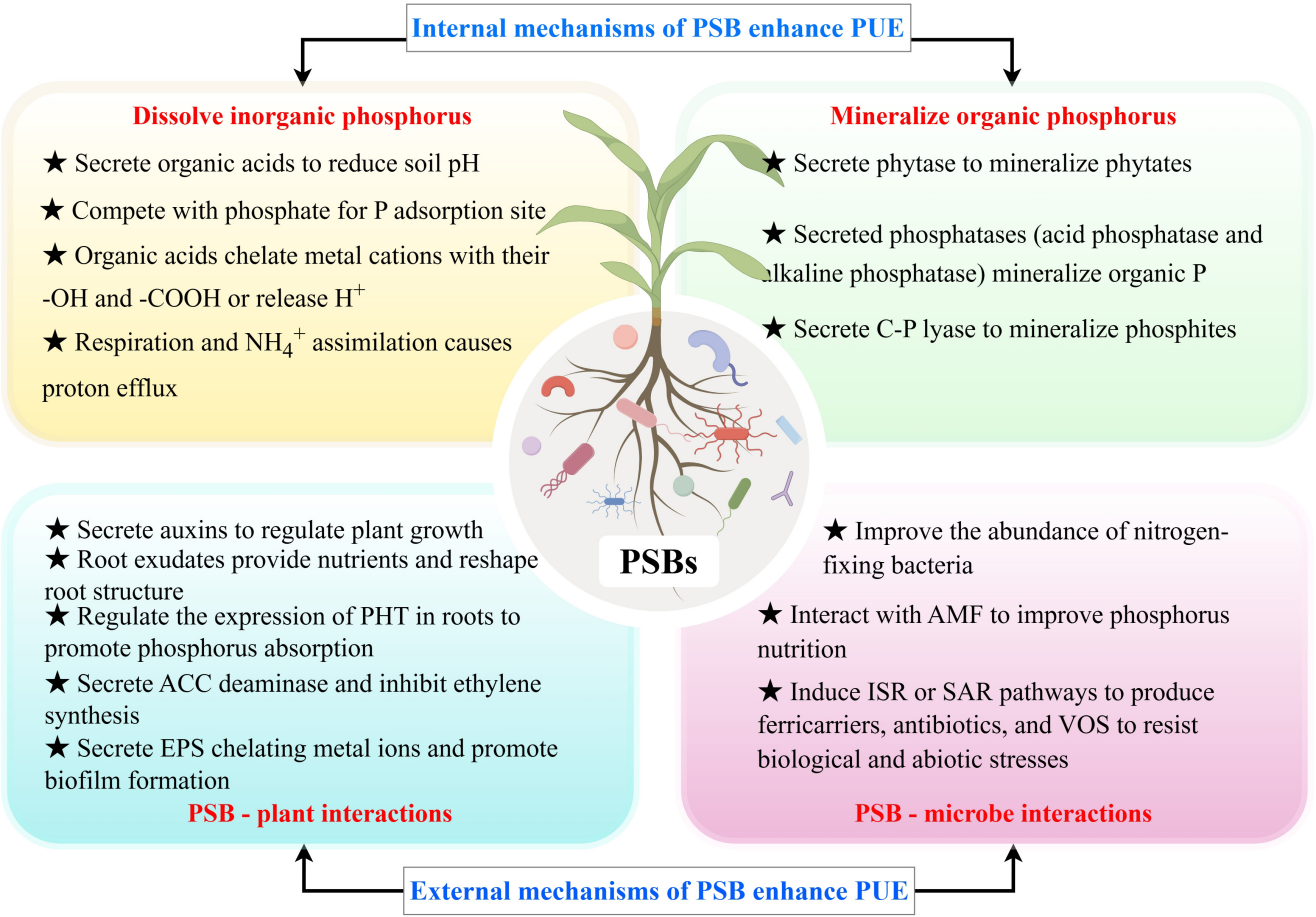
Biological mechanism of PSB promoting phosphorus uptake by crops.

Microorganisms play a crucial role in driving global biogeochemical cycles, converting organic matter into inorganic substances, and significantly influencing soil material turnover (Gao et al., 2024). As key members of the rhizosphere microbiome, PSB enhance P availability in soil by solubilizing both organic phosphate (Po) and inorganic phosphate (Pi) through multiple mechanisms (Figure 1). These include: (1) the secretion of acidic compounds that lower soil pH (Pang et al., 2024); (2) the Secrete a variety of enzymes that catalyze the mineralization of organic phosphorus (Akplo et al., 2025); (3) interactions with plant roots that stimulate the production of antimicrobial metabolites and volatile compounds (Upadhyay et al., 2023); and (4) the recruitment of beneficial soil microorganisms while outcompeting pathogens for nutrients and space, bolstering plant resilience (Maciel-Rodríguez et al., 2025; Wen et al., 2025).

### 3.1 Dissolution of inorganic P by acids

**FIGURE 2:**
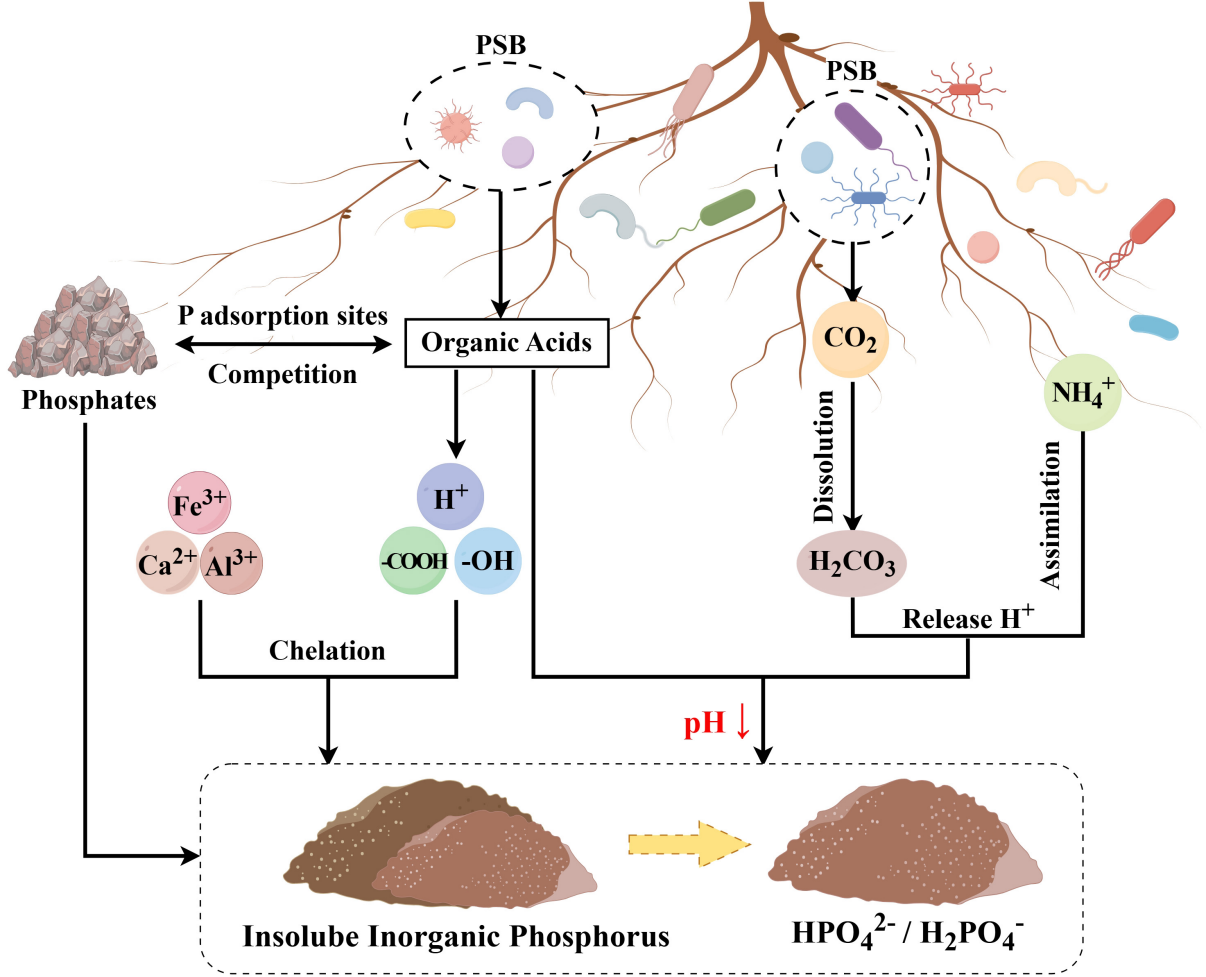
Mechanism of acid-mediated inorganic phosphorus solubilization by PSB.

The mechanisms through which PSB solubilize insoluble P include acidification, chelation, redox reactions, and polymer formation. These mechanisms are associated with the production of organic acids, protons (H^+^), and redox-active metals (Wei et al., 2018). Among them, it is widely accepted that the P solubilizing ability of PSB is linked to the release of low molecular weight organic acids (Wei et al., 2018). PSB primarily achieve the acid dissolution of inorganic P in soil through the following mechanisms (Figure 2). The primary mechanism of inorganic P solubilization is the secretion of low molecular weight organic acids, such as gluconic acid (GA) and 2-ketogluconic acid (Buch et al., 2008; Zeng et al., 2017). These organic acids release protons (H^+^) into the solution, directly acidifying the immediate microenvironment of the bacterial cells. This acidification, in turn, increases the solubility of insoluble phosphate salts [e.g., Ca_3_(PO_4_)_2_], leading to their dissolution (Luo et al., 2025). Second, organic acids secreted by PSB compete with phosphate ions for adsorption sites on soil particles, enhancing the solubilization of inorganic P and increasing its bioavailability at the root-soil interface (Demissie et al., 2013). Organic acids, such as citric acid, oxalic acid, and acetic acid, secreted by PSB, have organic acid anions compete with phosphate ions for adsorption sites on soil particles, thereby dissolving solid P and releasing it as plant-available soluble inorganic P (Rodríguez et al., 2006; Timofeeva et al., 2022; Wagh et al., 2014). Third, organic acids can chelate metal cations (e.g., Fe^3+^, Al^3+^, and Ca^2+^) that are bound to phosphate ions through their hydroxyl and carboxyl groups, thus further promoting the release of plant-available phosphate (Liu M. et al., 2025; Zhang et al., 2020). Fourth, protons are released when carbon dioxide (CO_2_) produced by biological respiration dissolves and forms carbonic acid (H_2_CO_3_); these protons can readily dissolve calcium apatite and promote the release of phosphate ions (Charana Walpola, 2012; Tian et al., 2021). The presence of NH_4_^+^ also lowers the pH, and its nitrification generates H^+^ (Khan et al., 2007; Zhu et al., 2018). It is therefore postulated that H^+^ excretion originates from both respiratory H_2_CO_3_ formation and NH_4_^+^ assimilation (Tian et al., 2021). This may serve as an alternative mechanism for lowering pH and dissolving mineral P when NH_4_-N is used as a nitrogen source, improving the bioavailability of P (Li H. P. et al., 2023).

Currently, research into the mechanisms of organic acid secretion has driven the development of various application technologies, including the creation of new biological fertilizers and soil amendments. While organic acid secretion is considered the primary P solubilization mechanism for PSB, the relationship between organic acid production and P solubilization efficiency is complex. Environmental factors, such as pH, temperature, oxygen concentration, and humidity, influence the physiological and metabolic state of PSB, thereby directly or indirectly affects the biosynthesis and extracellular secretion of organic acids, as well as the efficiency and rate of insoluble phosphate solubilization. Understanding the impact of these factors, along with microbial enzyme activity and soil physicochemical properties, will be essential for optimizing PSB-based solutions for P management.

### 3.2 Enzymatic hydrolysis of organic P

**FIGURE 3:**
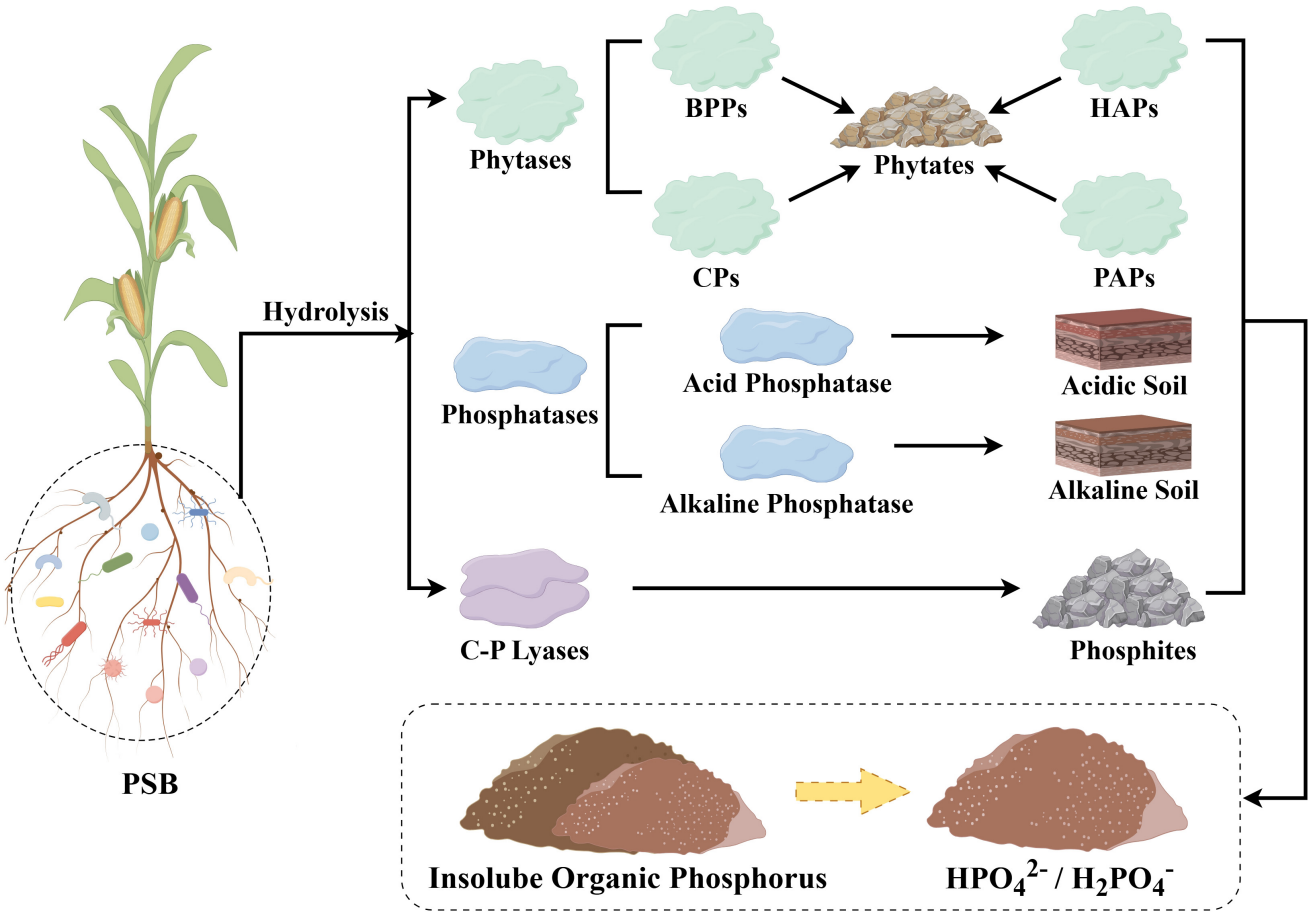
Enzymatic degradation mechanism of organic phosphorus by PSB.

Organic P constitutes a significant portion of the soil P, typically accounting for 30–50% of total P, with phytate (inositol phosphate) as its predominant form (Kumar and Shastri, 2017; Sharma et al., 2013). Phytate (inositol phosphate), characterized by an inositol core (a cyclic sugar alcohol) bound to 1–6 phosphate groups (Liu C. et al., 2025). PSB secrete various enzymes to mineralize organic P, which can be broadly categorized based on their catalytic mechanisms and the types of chemical bonds they target (Figure 3).

(1) Enzymes Catalyzing P-O Ester Bond Hydrolysis: These include phytase and phosphatase. Phytates often form complexes with cations or adsorb onto various soil organic components, making them less accessible for plant absorption (Azeem et al., 2015; Rawat et al., 2021; Singh and Satyanarayana, 2011). Studies have shown that phytase plays a key role in the mineralization of phytates (Rao et al., 2009). They degrade phytate (myo-inositol hexakisphosphate) into lower-order inositol phosphates and eventually release soluble P (Sharma et al., 2013). For example, *Pseudomonas corrugata* SP77 and *Serratia liquefaciens* LR88 exhibit high phytase activity, with P solubilization efficiencies of 714.96 and 306.74 μg/mL, respectively (Ben Zineb et al., 2020), the high phosphorus-solubilizing rate indicates their significant contribution to increasing plant-available phosphorus. In cereal crops, inoculation with PSB that produce phytase can enhance P uptake without the need for fertilizers (Martínez et al., 2015). In addition to phytase, PSB secrete various phosphatases, which can be classified as either acid or alkaline phosphatases, depending on their optimal pH values (Nannipieri et al., 2011). Alkaline phosphatase optimal pH is above 7, most often between 9 and 10 while acid phosphatase has a pH optimum between 4 and 6 (Elhaissoufi et al., 2022). Studies have shown that alkaline phosphatases can hydrolyze approximately 90% of the total organic P in soil, catalyzing the hydrolysis of P-O ester bonds and releasing inorganic phosphate (Pi) for plant utilization (He R. et al., 2025; Jarosch et al., 2015). Inoculation with PSB that produce both acid and alkaline phosphatases has been shown to significantly increase crop biomass, promote root growth, and enhance P uptake in pot experiments (Chen and Liu, 2019; Liu et al., 2023).

(2) Enzymes Catalyzing C-P Bond Cleavage: These include phosphonate hydrolases (C-P cleaving enzymes). These enzymes are membrane-bound complexes that catalyze reactions involving β-carbonyl electron-withdrawing groups, enabling the heterolytic cleavage of various types of phosphonates (alkyl, amino alkyl, and aryl phosphonates) (Kafarski, 2019), which results in the production of hydrocarbons and Pi. This is the primary mechanism by which microorganisms utilize phosphonates (Seweryn et al., 2015; Stosiek et al., 2020; Villarreal-Chiu, 2012).

As a result, the P solubilization mechanisms of PSB in soil are complex and diverse, encompassing the acid dissolution of inorganic P, enzymatic mineralization, hydrolysis of insoluble organic P, and metal cation chelation. In addition to these internal mechanisms, PSB also employ external regulatory strategies, indirectly facilitating the dissolution of insoluble P by modifying the structure and abundance of soil microbial communities.

### 3.3 Interaction between PSB and plant roots

**FIGURE 4:**
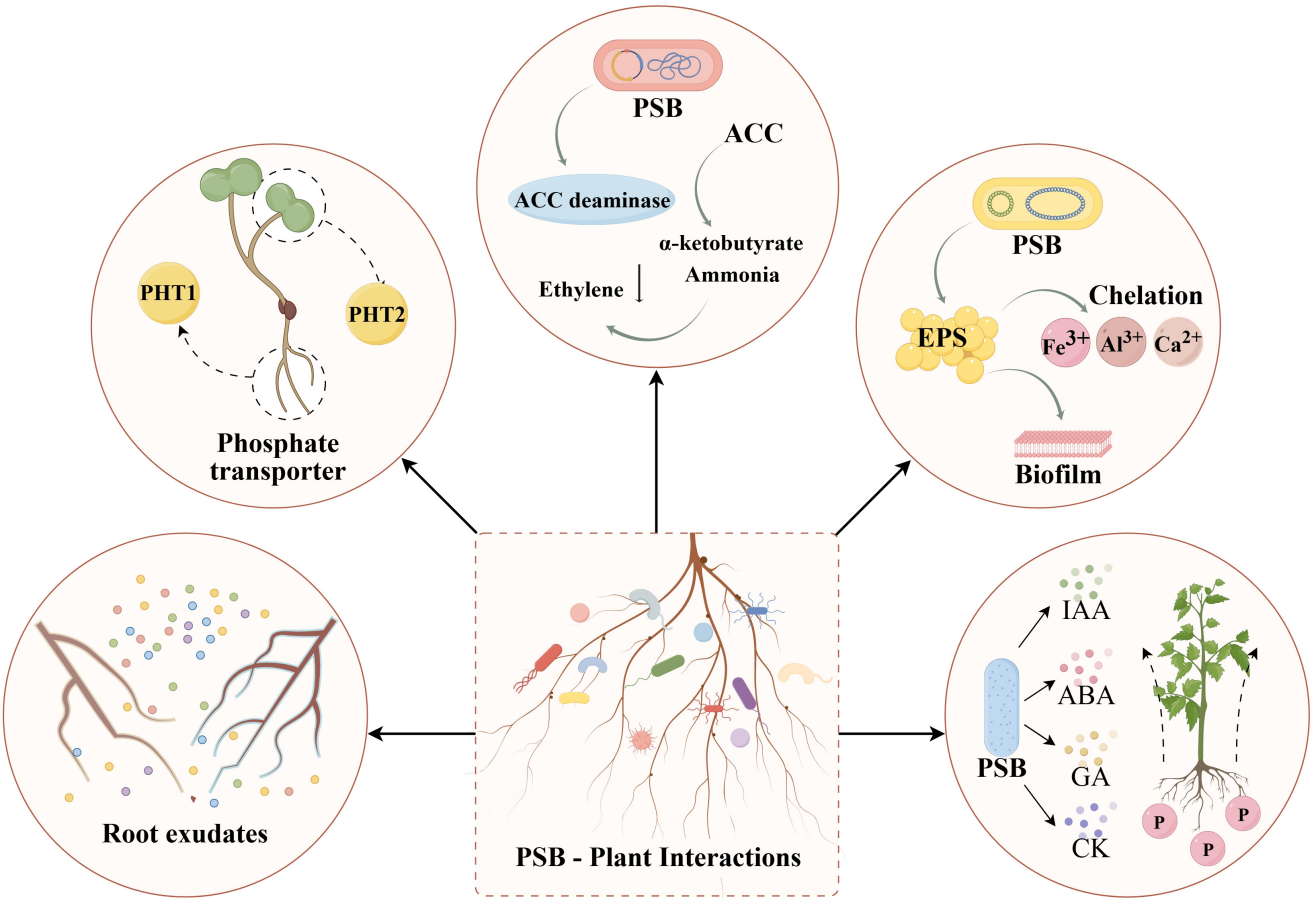
Interaction between PSB and plant roots.

PSB are capable of solubilizing P. This ability is influenced not only by the genetic traits of the bacteria but also by their interactions with plant roots (Figure 4). The internal mechanisms of PSB-root interactions mainly include: the regulation of root exudates, regulation of P transport protein expression, and the production of 1-aminocyclopropane-1-carboxylate deaminase, exopolysaccharides, and plant hormones.

#### 3.3.1 Regulation by root exudates

Plants release a variety of metabolites and secondary metabolites through their roots, particularly the fine roots, to regulate the rhizosphere environment during different growth stages (Ma et al., 2022). The primary metabolites in root exudates provide essential nutrients for rhizosphere microorganisms and influence the interactions between plant roots and microorganisms (Pang et al., 2021). Carbohydrates and amino acids are key substrates for rhizosphere microbial growth and are usually released in large quantities, which enables PSB to continue growing in the rhizosphere and alters the structure and abundance of the microbial community (Nwachukwu et al., 2021; Pongrac et al., 2020). For example, an increase in the concentration of root exudates, such as oxalate, malate, and citrate, can act as chemical attractants, attracting PSB and other soil microorganisms (Pascale et al., 2020; Tsai et al., 2020). In return, PSB promotes the uptake of plant P by regulating plant root system architecture (such as promoting the growth of plant root hairs), thereby enhance the absorption of nutrients by plant roots (Grover et al., 2021). Secondary metabolites, derived from primary metabolites or intermediates in primary metabolic pathways, are categorized into major families, such as phenolics, terpenes, steroids, alkaloids, and flavonoids, based on their biosynthetic pathways (Kessler and Kalske, 2018; Piasecka et al., 2015). These metabolites act as signals in plant-microbe symbiosis, altering the structure of host-associated microbial community and promoting plant growth and development (Guerrieri et al., 2019).

#### 3.3.2 Regulation of phosphate transporter expression (PHT1 and PHT2)

The absorption of phosphorus by plants needs to rely on phosphate transporter (Deng et al., 2017). Under P-deficient conditions, PSB regulates the expression of plant root phosphate transporters (PHTs) through complex gene regulatory networks and signaling molecules, enhancing plant P uptake (Timofeeva et al., 2022). Most plants express two types of phosphate transport proteins, PHT1 and PHT2, to maintain optimal P concentrations in cells (Bargaz et al., 2021). PHT1 is a high-affinity transport protein primarily expressed in roots, whereas PHT2 is a low-affinity transport protein responsible for P transport in plant tissues such as leaves, flowers, young fruits, and roots (Yang et al., 2020). It has been reported that PSB strains can regulate plant gene expression based on environmental conditions, such as soil P-deficiency. For example, *P. putida* can alleviate P deficiency stress in *Arabidopsis* by upregulating the expression of PHT1 and PHT2 (Srivastava and Srivastava, 2020). After inoculation with *Bacillus* spp., the expression of PHT1 in wheat roots increases, thereby promoting P absorption (Saia et al., 2015). Furthermore, PSB inoculation leads to the increased expression of inorganic phosphate transporters in plant roots, resulting in changes to root morphology, such as smaller roots, increased lateral roots, and enhanced root hair growth. These changes enhance P acquisition by increasing the surface area for nutrient absorption and stimulating phosphatase production to mobilize otherwise inaccessible P sources (Abbasi, 2023). Therefore, PSB play a crucial role in promoting efficient P absorption through the upregulation and downregulation of phosphate transport protein expression in plant roots.

#### 3.3.3 Secretion of 1-aminocyclopropane-1-carboxylate deaminase, exopolysaccharides, and plant hormones

Ethylene (ET) is a crucial plant hormone involved in regulating plant growth and development. Under stress conditions, ET production increases as an adaptive response, but excessive levels can inhibit overall plant growth (Gamalero and Glick, 2015; Khan et al., 2020). PSB can degrade ACC, the direct precursor of ET, into α-ketobutyrate and ammonia via the enzyme 1-aminocyclopropane-1-carboxylate deaminase (ACC deaminase), thereby lowering ET levels and alleviating plant stress (Orozco-Mosqueda et al., 2020; Paço et al., 2020). For example, *Pseudomonas* spp. is among the most extensively studied ACC deaminase-producing PSB (Glick and Nascimento, 2021). Additionally, PSB can promote root biomass and induce changes in root morphology through the secretion of ACC deaminase (Rawat et al., 2021). For example, the root biomass of rice is closely related to the level of ACC deaminase produced by *Alcaligenes* spp., with ACC deaminase positively correlated with root elongation (Bal et al., 2013).

Exopolysaccharides are high-molecular-weight carbohydrates produced by microorganisms (Netrusov et al., 2023). It has been shown that PSB strains that can synthesize EPS exhibit significantly higher phosphate solubilization activity compared to strains that do not produce EPS (Li H. P. et al., 2023). As a component of biofilms, EPS help PSB adhere to plant root surfaces, establish colonization, and absorb nutrients, thereby enhancing interactions with plants (Banerjee et al., 2021). For example, the EPS-producing *Paenibacillus polymyxa* GOL 0202 strain, which produces a high EPS content, exhibits a strong calcium phosphate solubilization ability. Inoculation with this strain significantly improves wheat seedling germination rates and root and shoot length (Cherchali et al., 2019). The EPS-producing *Enterobacter* sp. EnHy-401, *Arthrobacter* sp. ArHy-505, and *Azotobacter* sp. AzHy-510 display significantly higher tricalcium phosphate solubilization ability than non-EPS-producing strains such as *Enterobacter* sp. EnHy-402 (Yi et al., 2008) Under identical conditions, EPS-producing *Enterobacter* sp. EnHy-401 produces more organic acids and achieves greater phosphate solubilization than its non-EPS-producing counterpart (EnHy-402). This suggests that the synergistic action of EPS and organic acids is a key mechanism for accelerating the dissolution of phosphate minerals (Yi et al., 2008).

PSB can secrete a variety of plant hormones that regulate plant growth, including cell division and differentiation, organ development, fruit maturation, flowering, and seed formation (Wang et al., 2024). For example, *Bacillus tequilensis* can secrete abscisic acid, auxin (IAA), and gibberellins, which increase soybean stem biomass and chlorophyll content, and improve leaf structure under heat stress (Mukherjee et al., 2022). The IAA secreted by PSB acts synergistically with the plant’s endogenous IAA to regulate root morphology, growth, and nutrient uptake (Haidar et al., 2018). Auxin is one of the most common plant hormones produced by PSB. Many PSB genera, including *Pseudomonas*, *Mycobacterium*, *Rhizobium*, and *Bacillus*, can secrete IAA, which is crucial for plant-microbe interactions (de Lima et al., 2024). Tryptophan, a signal molecule between root nodules and host plants, triggers the synthesis of IAA in PSB through multiple pathways (Hassan and Bano, 2015). In addition to IAA, PSB can also secrete cytokinins, which promote root cell proliferation, leading to excessive lateral root and root hair growth and thereby improving the plant’s nutrient and water uptake (Timofeeva et al., 2022). The increased root surface area strengthens the interaction between PSB and plant roots, further promoting P mobilization (Timofeeva et al., 2022). Thus, PSB promote plant growth and help plants adapt to environmental stress by secreting ACC deaminase, exopolysaccharides, and plant hormones. This mutualistic relationship benefits plant growth and adaptation while providing a stable habitat and nutrient source for PSB.

### 3.4 Phosphate-solubilizing bacteria-microbe interaction mechanisms

**FIGURE 5:**
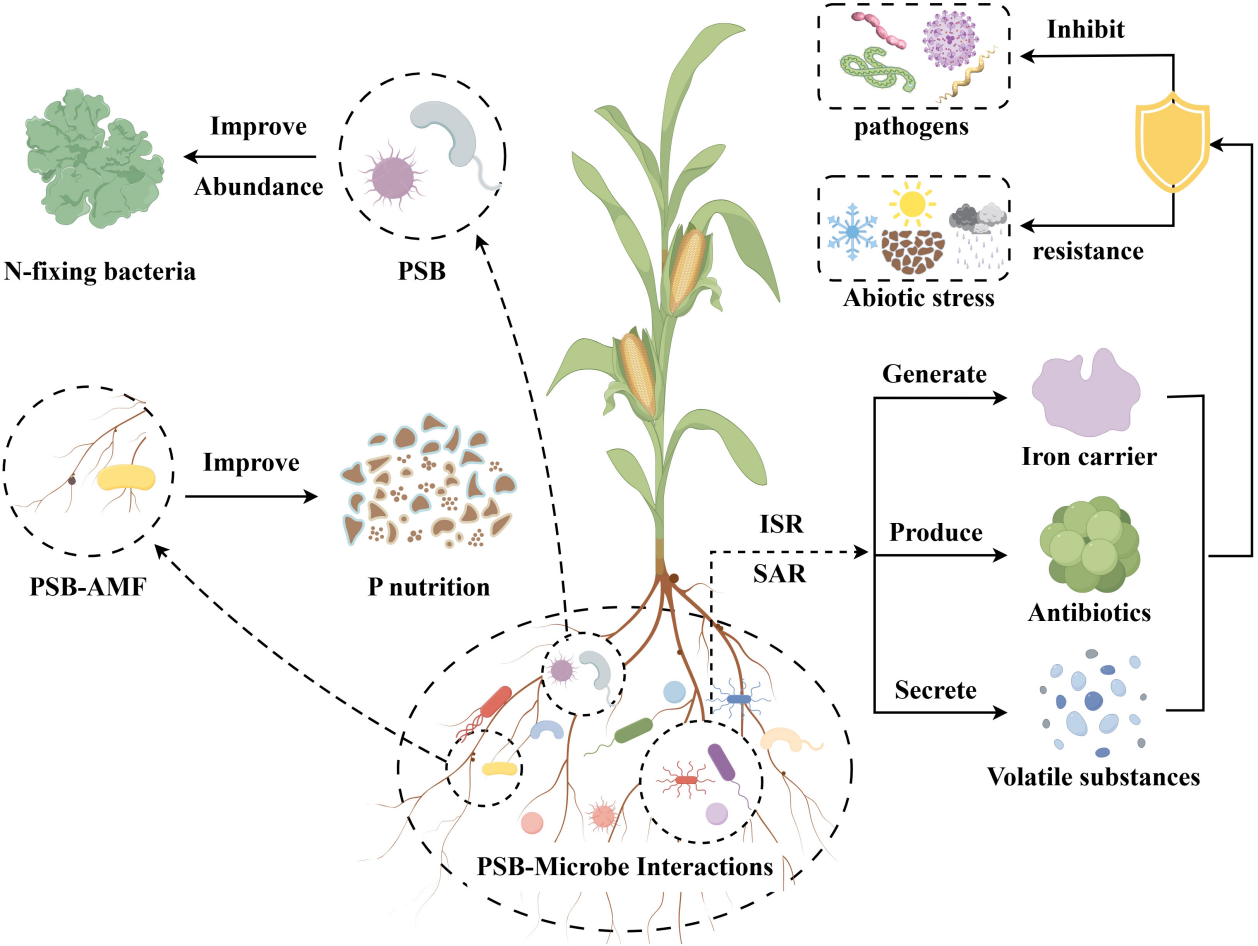
Interaction between PSB and microorganisms.

PSB optimize the rhizosphere environment by secreting a range of bioactive substances, such as organic acids, enzymes, plant hormones, and volatile organic compounds (VOCs) (Maciel-Rodríguez et al., 2025; Wen et al., 2025). Some of these secretions can provide nutrients for beneficial microorganisms and thus attract them (Zhu et al., 2025). In addition, active substances such as antibiotics secreted by PSB will inhibit the growth of pathogenic microorganisms. This regulatory action enriches the microbial diversity of the rhizosphere, enhances the community structure, and promotes the abundance and activity of microorganisms closely related to plant growth and development (Figure 5).

The primary strategy is to recruit beneficial microbial communities. The rhizosphere is home to a wide variety of microorganisms, making it an extremely complex environment. When PSB strains are applied to the rhizosphere, they can trigger interactions between the applied strains and other soil microorganisms. These interactions play a crucial role in regulating various chemical and biophysical processes in the rhizosphere, collectively promoting plant growth (Gupta et al., 2022; He C. et al., 2025). For example, the inoculation of composite formulations containing multiple PSB strains significantly increases the abundance of nitrogen cycling genes in the rhizosphere, as well as the abundance of *Betaproteobacteria* and *Chitinophagia*. Significant changes in the abundance of *Deltaproteobacteria* and *Gemmatimonadetes* have also been reported with an increase in soil-available P and phosphatase activity (Xing et al., 2022). These bacteria have been shown to improve ammonia oxidation and denitrification processes in the soil, promote the decomposition of complex organic matter, enhance the microbial utilization of rhizodeposits, and increase the activity of plant-growth-promoting bacteria (Bellini et al., 2018). Furthermore, arbuscular mycorrhizal fungi (AMF), the most common symbionts that enhance plant P uptake, play a critical role in maximizing plant P acquisition (Liu et al., 2024). The external hyphae of AMF can absorb P and transfer it to plants. When co-inoculated with PSB, the solubilization activity of PSB is effectively enhanced, improving the efficiency of P absorption in plants under various conditions, thereby maximizing the inoculation effect (Liu et al., 2024). In P-deficient soils, AMF can contribute up to 50-75% of the plant’s P uptake while consuming 5-30% of the photosynthetic products (Mai et al., 2019). Some studies have reported synergistic effects on plant growth, P absorption, and soil P solubilization when AMF and PSB are co-inoculated under both normal and stressed conditions (El Maaloum et al., 2020; Zai et al., 2021). Additionally, exudates from AMF (such as sugars, carboxylic acids, and amino acids) can stimulate the growth of PSB, thereby enhancing their viability in the soil environment post-inoculation (Tian et al., 2021).

The second strategy is to enhance resistance to pathogenic microbial communities. By leveraging various metabolites and volatile compounds, PSB play a pivotal role in modulating the composition of the soil microbial community. Through the release of antagonistic substances in the rhizosphere, these bacteria impede soil pathogens, thereby improving soil health and offering an effective alternative to traditional pest control methods (Chandran et al., 2021; Khoshru et al., 2020; Wang et al., 2024).

PSB can produce antibiotics, hydrolytic enzymes, and antimicrobial compounds that suppress the growth of pathogens, thereby protecting plants from infection. In addition, antibiotics secreted by PSB can also indirectly protect plants from pathogens, viruses, and fungi by inducing systemic resistance (ISR) and systemic acquired resistance (SAR) responses of host plants (de Andrade et al., 2023; Khoshru et al., 2023; Sibanyoni et al., 2025). Antibiotics are low-molecular-weight, multivalent microbial-derived substances, classified into volatile and non-volatile compounds (Beneduzi et al., 2012). These antibiotics provide direct resistance to pathogens and also promote disease suppression in plants through the induction of systemic resistance. The various beneficial bacterial genera in PSB can produce antibiotics as an effective defense mechanism against pathogen invasion. Antibiotic production is a major mechanism by which PSB protect plants from biotic stress and promote plant growth and metabolic activities (Beneduzi et al., 2012; Dutta et al., 2022). For example, *Bacillus*, a dominant genus in PSB, produces various antibiotics, including bacillomycin, rhizomycin and actinomycin. It can also produce antimicrobial surfactants to effectively combat plant pathogens (Khoshru et al., 2023). In addition, *Bacillus* produces iron carriers and exopolysaccharides that help regulate ion balance, synthesize microbial metabolites, and control plant disease threats (Wang et al., 2024). Furthermore, *P. fluorescens*, an effective biological control agent, also has the potential to protect plants from pathogen attacks (Lucas et al., 2017). Hydrolytic enzymes secreted by PSB protect plants from pathogens and include cellulases, proteases, chitinases, and lipases (Zou et al., 2023). These enzymes primarily work by hydrolyzing polymeric compounds, breaking down the cell walls, proteins, and DNA of pathogens (Yu et al., 2024). Therefore, the application of exogenous beneficial microbes in biofertilizers can stimulate the accumulation of beneficial soil microorganisms, forming a beneficial microbial community to counteract pathogens and ultimately recruit more disease-resistant microorganisms.

## 4 Application of phosphate-solubilizing bacteria formulations

Studies have shown that bioformulations can reduce chemical fertilizer use by 30% while increasing crop yields by 30% (Elhaissoufi et al., 2022). In recent years, many countries, including China, have developed various biofertilizers based on PSB (Cheng et al., 2023). For instance, in calcareous Takhli soils of Thailand, the application of phosphate-solubilizing bacterium *Bacillus* spp. as a biofertilizer increased available phosphorus by approximately tenfold (reaching 30 mg L^−1^), and when combined with chemical fertilizer at 225 kg ha^−1^, it also enhanced fresh sugarcane yield by 69%, sugar yield by 99%, and commercial cane sugar (CCS) content by 18% (Chungopast et al., 2021). Common PSB formulations include solid and liquid formulations, which have been widely applied in the cultivation of crops such as legumes, cereals, and solanaceous plants, leading to significant increases in crop production (Table 2).

**TABLE 2:** Examples of studies reporting beneficial effects of solid and liquid bio-formulations on various crops.

Formulation types	Plants	Strains used	Effects of formulation on plants	References
SOLIDE	*Wheat*	*Bacillus velezensis* BV9	Significantly increase the plant growth factors, reduce the disease by > 50 %	(Moradi Pour et al., 2024; Pour et al., 2022)
		*Ochrobactrum* sp. SSR *Enterobacter* sp. ZW9 *Enterobacter* sp. ZW32	Improve the wheat growth and yield under P-deficient conditions	(Yahya et al., 2022)
	*Phaseolus vulgaris L.*	*Sinorhizobium mexicanum* ITTG R7^T^ *Rhizobium calliandrae* LBP2-1^T^ *Rhizobium etli* CFN42^T^	To augment nitrogenase activity and nodulation capacity in leguminous flora	(Ruíz-Valdiviezo et al., 2015)
		*Bacillus subtilis* Vru1	Protect the biocontrol bacteria against harmful environmental conditions and to enhance their survival rates, controlling of Rhizoctonia solani infection in bean plants.	(Saberi-Rise and Moradi-Pour, 2020)
	*Cotton*	*Raoultella planticola* Rs-2	Be beneficial for seed germination and seedling development under salinity stress	(Wu et al., 2014)
	*Arachis hypogea L.*	*Azospirillum brasilense strain* TNAU *Pseudomonas fluorescens strain* PF1	Promot seedling growth of groundnut, enhance the tap root growth, stimulate the development of plant lateral roots	(Prasad and Babu, 2017)
	*Blue maize*	*Pseudomonas putida* KT2440 *Sphingomonas* sp. OF178 *Azospirillum brasilense* Sp7 *Acinetobacter* sp. EMM02	Increased the shoot and root dry weight, plant height and plant diameter of crops	(Molina-Romero et al., 2017)
	*Maize*	*Bacillus cereus sensu lato strain* B25	Controll *F. verticillioides* in greenhouse assays, as well as eight other maize phytopathogenic fungi in vitro	(Martínez-Álvarez et al., 2016)
LIQUIDE	*Vitis vinifera* L. *grape*	*Pseudomonas putida* Rs-198	Promote alkaline phosphatase and invertase activity, increased the amount of available phosphorus, and enhanced the growth and quality of grape.	(Lu et al., 2020)
	*Stevia rebaudiana*	*Bacillus safensis* STJP	To augment growth, stevioside content, and nutrient uptake in Stevia rebaudiana	(Prakash and Arora, 2019)
	*Tomato*	*Beauveria bassiana* (B2 and TBb8)	Reduced the incidence of fruit borer in tomato, significant increase in growth parameters and yield, increased accumulation of defense related enzyme was observed in tomato plants	(Lakshmidevi et al., 2020)
	*Rice*	*Pseudomonas fluorescens strain* (Pf1, TDK1, and PY15)	Reduced the incidence of leaffolder pest in rice plants	(Saravanakumar et al., 2007)
	*Wheat*	*Paraburkholderia tropica* MTo-293	Increase the average values of the yields of wheat	(Bernabeu et al., 2018)
	*Vigna mungo* (L.) *Hepper*	*Bacillus* sp., *Streptomyces* sp., *Azotobacter* sp., *Frauteria* sp.	Increase the germination percentage, growth, and yield parameters of black gram	(E Leo Daniel, 2011)

Recently developed technologies to improve the inoculation efficiency of bioformulations include: (1) co-cultivation and co-immobilization of microorganisms, (2) selecting reliable functional carriers such as natural carriers (peat, compost, vermiculite, perlite, sludge) and polymeric carriers (alginate), and (3) developing new production techniques, such as biofilm fertilizers and nanoparticle formulations (Lobo et al., 2019). Whether preparing liquid or solid PSB formulations, the selection of an appropriate carrier material is crucial. For a carrier material to be considered suitable it should be: non-toxic to plants, microorganisms, humans, and the environment; easy to sterilize and process; environmentally friendly and renewable; readily available in large quantities; and cost-effective. Only with these characteristics can a formulation be practical and commercially viable during the inoculation process.

Solid and liquid formulations are widely used in different areas of agricultural production due to their unique application characteristics. The preparation of solid formulations typically involves inorganic or organic carriers such as granules, wettable powders, or dust. Various application methods can be applied for solid formulations prepared with different carriers (Mastan et al., 2019). Compared with solid formulations, liquid formulations have several advantages in practical inoculation processes, including a longer shelf life, higher microbial vitality, reduced risk of contamination, and better agronomic benefits (Vendan and Thangaraju, 2007). In practice, the appropriate formulation should be selected based on specific conditions to maximize the agronomic benefits of biofertilizers.

Additionally, the dual application of PSB with rock phosphate (RP) or organic fertilizers has been shown to significantly improve the P nutrition of cereal and leguminous crops (Shahzad et al., 2017). The combined use of PSB with RP or organic fertilizers creates a synergistic effect, leading to the production of high-efficiency P-based biofertilizers (Marra et al., 2022). The combination of PSB with rhizobia has also become a hot research topic in biofertilizer development. Co-inoculating PSB and rhizobia can increase nodulation rates by 42%, while also improving lentil germination rates and seed yield (Paliya et al., 2019). Therefore, compared to the application of a single microbial fertilizer, the synergistic use of multiple beneficial microorganisms leads to enhanced plant growth promotion and greater economic benefits.

## 5 Conclusion and future perspectives

This paper provides a detailed explanation of the role of PSB in enhancing crop P utilization efficiency through acid production and enzymatic activity. It introduces the biological mechanisms of PSB-plant root-microbe interactions, revealing how PSB can promote P release in the soil and alter microbial community structures. Additionally, the main application areas of PSB formulations and the key factors that influence their preparation and effectiveness were reviewed. However, despite the significant potential of PSB in improving soil P utilization, many challenges remain in their practical application.

Future studies should focus on improving PSB formulations and optimizing application technologies to further explore their potential in agricultural production and ecosystem management. Specifically, attention should be given to the following aspects:

The impact of the environment on PSB diversity and characteristics: While many PSB strains have been isolated and identified, systematic studies of how different soil types affect PSB distribution and functional characteristics are lacking. Furthermore, studies have revealed performance discrepancies of PSB between laboratory conditions and field applications, leading to limitations in their practical use. The underlying reasons for this phenomenon, however, remain insufficiently understood. Future research should employ integrated multi-omics analysis and molecular ecology approaches to conduct in-depth investigations into the diversity, abundance, and function of phosphate-solubilizing microorganisms. This will further elucidate their ecological roles and functional mechanisms within soil ecosystems, and decipher the epigenetic regulatory switches governing their environmental adaptability.

Optimization of formulation preparation processes. The preparation processes for PSB formulations still have limitations, such as issues with the stability, activity, and effectiveness of the formulations. Future efforts should focus on optimizing the preparation processes, including strain selection, cultivation conditions, and additive choices. Integrating microbiome engineering and synthetic biology could help design and construct engineered strains with enhanced P-solubilizing capabilities. By regulating the expression of P-solubilizing genes (e.g., overexpression of *gcd* for gluconic acid production) and optimizing metabolic pathways, as well as the use of some emerging technologies (such as nano-encapsulation of PSB, CRISPR-edited strains), the P-solubilizing efficiency and stability of PSB can be improved, providing technological support for the preparation and application of bioformulations.

Research on integrated fertilization models. As a biofertilizer, PSB formulations play a critical role in integrated fertilization models, which combine biofertilizers, chemical fertilizers, and soil amendments. Such integrated approaches are vital for improving soil fertility and increasing crop yields. Therefore, future studies should prioritize a multidisciplinary integration of PSB with complementary fertilization strategies. This involves combining PSB with precision agriculture tools (e.g., variable-rate technology and drone-based monitoring) and incorporating IoT-based real-time soil sensing systems. Such an integrated approach will enable data-informed management strategies to optimize inoculant and nutrient application. This synergy is essential for developing scalable and environmentally adaptive fertilization frameworks that promote sustainable agricultural productivity and long-term soil health.
